# Review for the generalist: evaluation of pediatric hip pain

**DOI:** 10.1186/1546-0096-7-10

**Published:** 2009-05-18

**Authors:** Kristin M Houghton

**Affiliations:** 1 K4-123 ACB BC Children's Hospital, 4480 Oak Street, Vancouver V6H 3V4, Canada

## Abstract

Hip pathology may cause groin pain, referred thigh or knee pain, refusal to bear weight or altered gait in the absence of pain. A young child with an irritable hip poses a diagnostic challenge. Transient synovitis, one of the most common causes of hip pain in children, must be differentiated from septic arthritis. Hip pain may be caused by conditions unique to the growing pediatric skeleton including Perthes disease, slipped capital femoral epiphysis and apophyseal avulsion fractures of the pelvis. Hip pain may also be referred from low back or pelvic pathology. Evaluation and management requires a thorough history and physical exam, and understanding of the pediatric skeleton. This article will review common causes of hip and pelvic musculoskeletal pain in the pediatric population.

## Background

Children who have hip pathology may present with a variety of non-specific symptoms. Transient synovitis and septic arthritis have similar early symptoms with the spontaneous onset of progressive hip, groin, or thigh pain; limp or inability to bear weight; fever; and irritability. Untreated intra-articular infection can lead to a permanent loss of hip function making it extremely important to differentiate possible infection from benign cases of transient synovitis. Other causes of hip pathology are often related to skeletal maturity and are fairly specific to the age of the child. An awareness of developmental anomalies as well as variation in skeletal maturation will aid the physician in evaluation and management. This article will review common musculoskeletal causes of hip and pelvis pain in the pediatric population.

### Clinical history

Children who have hip pathology may present with pain, refusal to bear weight, limp, or decreased movement of the lower extremity. If pain is present it is important to determine where it is coming from, as pelvis and low back pathology may refer pain to the hip region and hip pathology commonly presents with referred thigh or knee pain. Intra-articular hip pathology is usually localized to the groin. Once the pain is localized to the hip region, there is a limited differential diagnosis based on age and presentation. Diagnostic imaging is then useful to confirm the diagnosis.

The clinical history should include a thorough description of the pain characteristics (location, character, onset, duration, change with activity or rest, aggravating and alleviating factors, night pain); trauma (acute macrotrauma, repetitive microtrauma, recent/remote); mechanical symptoms (catching, clicking, snapping, worse during or after activity); systemic symptoms (fever, irritability); inflammatory symptoms (morning stiffness); neurological symptoms (weakness, altered sensation); gait (limp, altered weight bearing); effects of previous treatments (including antibiotics, analgesics, anti-inflammatories, physiotherapy) and the current level of function of the child.

A history of previous injury or surgery, neurological disorder, chronic inflammatory joint disease or bleeding diathesis, as well as diseases associated with spodyloarthropathies including psoriasis, acute uveitis and inflammatory bowel disease is significant.

### Anatomy

**Figure 1:**
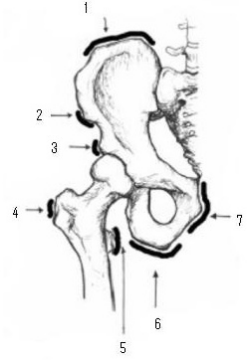
**Hip and Pelvis Anatomy**. 1 = Iliac crest (abdominal muscle attachment), 2 = Anterior superior iliac spine (sartorius attachment), 3 = Anterior inferior iliac spine (rectus femoris attachment), 4 = Greater trochanter (gluteal attachment), 5 = Lesser trochanter (psoas attachment), 6 = Ischial tuberosity (hamstring attachment), 7 = pubic symphysis and inferior pubis ramus (gracilis and adductor attachments).

The basic anatomy of the hip with muscle attachments is shown in Figure 1. The hip joint (acetabulofemoral joint), sacroiliac (SI) joint and pubic symphysis make up the pelvic girdle. The hip joint is a ball and socket articular joint and the pubic symphysis is a fibrous joint. The SI joint is a diarthrodial joint with hyaline cartilage on the sacral side and fibrocartilage on the iliac side. It also contains numerous ridges and depressions, indicative of its function for stability more than motion. The pubic symphysis and SI joint are very stable joints with limited motion whereas the hip joint is mobile in multiple planes. Maximal intracapsular volume of the hip is possible with the hip in a position of flexion, abduction, and external rotation; this is the position of comfort in children with hip effusions. The superficial muscles of the hip and pelvis region can be grouped into four quadrants based on their position and function: anterior flexor group (iliopsoas, sartorius, rectus femoris), medial adductor group (gracilis, pectineus, adductor muscles), posterior extensor group (hamstring muscles, gluteus maximus) and lateral abductor group (gluteus medius and minimus).

The hip joint has two separate development patterns and the level of skeletal maturity has an effect on injury patterns and disease processes. Acetabular growth occurs from the triradiate cartilage and cartilaginous periphery of the acetabulum and normal development results in a congruent, stable joint. Injuries or disruption of the developing triradiate cartilage may cause acetabular dysplasia and degenerative joint disease later in life.[1] The proximal femur has three ossification centres: the capital femoral epiphysis and the greater and lesser trochanters. The hip and pelvis have several apophyses which allow circumferential growth. These secondary sites of ossification also have muscle attachments and avulsion injuries are more common than musculotendinous injuries in skeletally immature individuals.

### Physical Examination

The general physical examination should include temperature and vital signs. During the musculoskeletal assessment the physician should try to reproduce the patient's pain through palpation and manipulation. This should include an evaluation for genetic predisposing factors such as excessive stiffness, joint laxity and/or increased or decreased muscle tone. Functional biomechanics should be assessed by evaluation of gait and maneuvers such as squatting, single leg hopping, lunging, zig-zag running and sit-ups. Most causes of hip pain are unilateral, allowing comparison to the unaffected side. The lumbar spine, SI joint, knee and abdomen should always be examined. A complete musculoskeletal exam to look for joint swelling should be done if there is a history of inflammatory symptoms. It should be noted that it is exceptionally rare to appreciate swelling of the hip on physical exam as it is a deep joint.

#### Observation

##### Standing

Pelvis heights, degree of lumbar lordosis and lower limb alignment are assessed with the patient standing. Both anterior superior iliac spines (ASIS) and posterior superior iliac spines (just below the dimples of Venus) should be in the same horizontal plane; if they are not there may be pelvic obliquity. An exaggerated lumbar lordosis may be due to weak abdominal muscles or hip flexion contracture.

##### Supine or sitting

Lower limb lengths and alignment can be assessed in supine or sitting position.

##### Gait

In normal walking, 60% of time is spent in the stance phase (20% double stance) and 40% in the swing phase.[2] During gait, the pelvis and trunk usually shift slightly to the weight-bearing side. If there is gluteus medius weakness, the lateral shift of the trunk is accentuated ("abduction or gluteus medius lurch") and if there is gluteus maximus weakness the patient will push their thorax posteriorly to maintain hip extension ("extensor or gluteus maximums lurch").[2] Altered gait may be due to truncal or lower limb malalignment, lower extremity muscle weakness, joint instability, joint limited range of motion, and/or pain.

#### Palpation

Patients with intraarticular pathology usually have pain in the groin. Surface anatomy for anterior structures is best appreciated with the patient standing and surface anatomy for posterior and lateral structures with the patient lying on their side with their hip flexed. It is important to palpate specific structures. The point of maximal tenderness should be correlated with the underlying bone or soft tissue anatomy. Palpate and note tenderness over the anterior superior iliac spines (ASIS), iliac crest, iliac tubercles, greater trochanter and pubic tubercles with the patient standing. Palpate the posterior superior iliac spines (just below the dimples of Venus), greater trochanter, ischial tuberosity and SI joint region with the patient lying on their side with their hip flexed. Palpate along the course and attachment of ligaments and tendons. Palpate for inguinal lymph nodes.

#### Range of motion

Active movements. Hip flexion (most children can bring their knee to touch their chest; 120–135°), hip extension (30°), abduction (45–50°), adduction (20–30°).

Passive movements. Rotation measured with hip and knee flexed to 90°. Total internal and external rotation should equal 90°, internal rotation is greater with femoral anteversion and external rotation is greater with femoral retroversion. Femoral anteversion decreases with age (30° to 15°).[3] If hip range of motion is normal in single planes, combined movement in the hip quadrant position (flexion, adduction, internal rotation) may reproduce pain, especially if there is intra-articular pathology as these movements decrease intracapsular volume.

#### Special tests

Thomas test for flexion contracture. Patient supine with level pelvis – place your hand under the patient's lumbar spine and flex one hip as far as possible. The patient's back should flatten and the extended leg should remain straight. A hip flexion contracture is present if the patient is unable to extend the leg straight without arching the spine. The degree of contracture is measured as the angle between the table and the leg at the point of greatest extension.

Trendelenburg Test. Positive when patient stands on one leg and the contralateral hip drops, indicative of gluteals/hip abductor weakness.

Ober test for contraction of iliotibial band. Patient lying on their side with their affected leg uppermost. Abduct the leg and flex the knee to 90° while keeping the hip joint in neutral position and then bring the leg into slight hip extension and external rotation. On release of the leg the thigh should drop into an adducted position. The thigh will remain abducted in a positive test.

FABER or Patrick test. Patient lying supine and leg passively brought into Flexion, ABduction, External Rotation with foot resting on opposite knee. Press down gently but firmly on the flexed knee and the opposite anterior superior iliac crest. Pain may be felt in the groin with intraarticular hip pathology or SI joint region with SI pathology.

#### Flexibility

The hip should be moved thru active range of motion and then placed thru full passive range of motion. Muscles that span two joints are important for functional range of motion and should be tested independently (rectus femoris, biceps femoris).

#### Strength

Hip flexion [iliopsoas, sartorius, rectus femoris]

Hip extension [hamstring muscles, gluteus maximus]

Hip abduction [gluteus medius and minimus]

Hip adduction [gracilis, pectineus, adductor muscles]

Abdominal flexion/resisted sit-up – [rectus abdominis].

#### Lumbar Spine and knee joint exam

Range of motion. Further detailed exam as appropriate.

#### Neurovascular exam

Patellar reflex [L2, L3, L4].

Sensation of the leg in major dermatomes.

Femoral artery pulsations.

### Investigations

Laboratory tests are necessary to exclude septic arthritis in young, febrile or unwell appearing children and adolescents. CBC, ESR or CRP, blood and joint cultures should be done if infection is suspected. Cultures of joint and synovial fluid should be sent in BACTEC culture bottles to optimize detection of clinically significant microorganisms.[4] Arthritis is a clinical diagnosis; anti-nuclear antibody (ANA), rheumatoid factor and HLA-B27 are helpful in classification and treatment but not diagnosis. CBC and peripheral smear should be done if malignancy is suspected.

### Diagnostic Imaging

Radiographs, ultrasound and magnetic resonance imaging (MRI) are the most common imaging tools used to assess the pediatric hip. Typically the first line of imaging investigation has been hip radiographs and ultrasonography. As with all tests, false positive and negative results may occur and clinical correlation is necessary.

#### Radiographs

Children and adolescents with hip pain, referred pain to the thigh or knee or limp require visualization of the proximal femur in two planes. Anterior posterior (AP) plain films of both hips, preferably taken with the patient standing and "frog leg" view are standard.[5]

#### Ultrasound

Ultrasonography can be used to identify an effusion, although it lacks specificity regarding the underlying disease. Ultrasound also can be used to guide aspiration of the hip joint.

#### Computed tomography (CT)

Computed tomography can detail bony anatomy, but exposes the patient to a moderate amount of radiation. Both CT and MR provide good ability for multiplanar imaging. CT has the advantage of being able to be manipulated to make 3D reconstructions.

#### Magnetic Resonance Imaging (MRI)

MRI provides increased soft tissue contrast and more detailed evaluation of articular and physeal cartilage, subchondral bone, periosteum, synovium and bone marrow elements.

The results from a study of fifty consecutive children presenting with acute non-traumatic hip pain indicate good sensitivity and specificity for MRI in the investigation of acute hip pain in children and suggest it is a more accurate method than ultrasonography and radiography (where routine aspiration of effusions is not performed).[6] Intravenous injection of gadolinium is not routine but is useful to delineate synovial enhancement.[7] Studies in adults have shown MR arthrograms have high specificity in detecting labral pathology or loose bodies but poor sensitivity.[8]

#### Technetium bone scan

Bone scan identifies areas of increased osteoblastic activity and can help localize infection and subtle areas of bone injury such as early stress fracture.

### Common musculoskeletal causes of hip and pelvis pain

Transient synovitis and septic arthritis are the most common diseases among young children with acute hip pain. Hip pain may be caused by conditions unique to the growing pediatric skeleton including Perthes disease, slipped capital femoral epiphyses and apophyseal avulsion fractures of the pelvis.

### Developmental conditions

#### Perthes disease

Perthes disease, an idiopathic avascular necrosis/osteonecrosis of the femoral epiphysis, usually affects 4 to 10 year olds, peaking between 5 and 7 years. It affects about four boys for each girl affected and is bilateral in 10%.[9] Children usually present with a limp or pain in the hip, thigh or knee. Examination of the knee is normal but there is limited and painful rotation and abduction of the ipsilateral hip. Internal rotation is usually affected more than external rotation. Trendelenburg may be positive. Radiographs vary with the stage of the disease but may show evidence of bone necrosis, fragmentation, reossification or remodeling and healing. The earliest signs include decreased size or increased density of the proximal femoral epiphyses on an AP view and crescent sign (a subchondral fracture that correlates with the extent of necrosis) on the lateral view. Bilateral Perthes disease is usually asynchronous; apparently synchronous bilateral Perthes' should raise the suspicion of an alternative diagnosis, such as epiphyseal dysplasia. Treatment consists of rest from aggravating activities and range of motion exercises. Occasionally orthoses or surgery may be required. Prognosis is largely dependent on the amount of femoral head involved, with a recent study citing femoral head involvement of more than 50% as the strongest predictor of poor outcome.[10] Age greater than 6 years also conferred worse prognosis than younger children.[10] The Herring or lateral pillar classification also strongly correlates with outcome. In Lateral Pillar Group A there is no loss of height in the lateral 1/3 of the head and little density change; in Lateral Pillar Group B, there is lucency and loss of less than 50% of the lateral height; in the B/C border group there is loss of 50% of the original height of the lateral pillar and in Lateral Pillar Group C, there is more than 50% loss of lateral height.[11] A prospective multi-center study with follow up at skeletal maturity on 345 patients with 337 affected hips found that children aged 8 years (6 years skeletal age) or older at onset and lateral pillar B group or B/C border group had better outcome with surgical treatment than with non-operative treatment. Children under age 8 at onset with Group B hips had a good outcome regardless of treatment and children with Group C hips had poor outcome, regardless of treatment.[12] The main long-term concern is early osteoarthritis.

Osteonecrosis of the proximal femoral epiphyses may also occur following trauma or may be associated with systemic disease (leukemia, lymphoma, systemic lupus erythematosus), hemoglobinopathies, coagulopathies, and as a complication of corticosteroid treatment.

#### Slipped Capital Femoral Epiphyses (SCFE)

**Figure 2:**
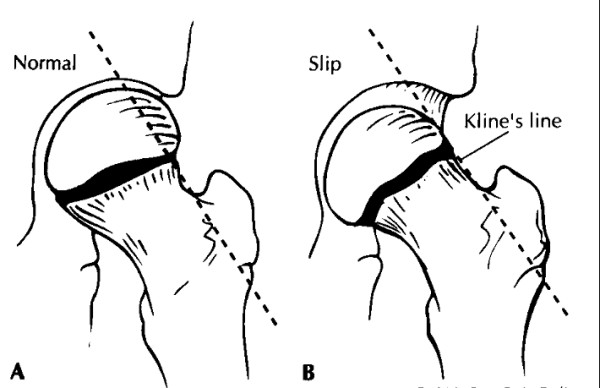
**Klein's line in normal situation versus in slipped capital femoral epiphysis **[13]. Klein's line is drawn along the radiographic border of the neck of the femur. This line should intersect the epiphysis. A, Klein's line in normal situation. B, Alignment of Klein's line with slip: the epiphysis is out of alignment.

SCFE, displacement of the proximal femoral epiphysis off the femoral neck, usually affects 11 to 14 year olds, is more common in obese children and boys and is bilateral in 20–40%.[13] Adolescents usually present with a limp and may have hip, groin or knee pain. The knee exam is normal. The hip is often preferentially held in abduction and external rotation with decreased active and passive internal rotation, flexion and abduction. Gait may be remarkable for an asymmetric outward foot progression angle and Trendelenburg may be positive. Patients are classified as having a stable or unstable slip based on their ability to weight bear on the affected leg. [14] Radiographs (AP and frog leg) of the hip may show widening and irregularity of the physis with posterior inferior displacement of the femoral head. On the AP view, a line drawn from the superior femoral neck (Klein's line) should intersect some portion of the femoral head. (Figure 2).

SCFE may compromise the vascular supply to the femoral head and lead to avascular necrosis; all cases warrant urgent orthopedic referral, and unstable SCFE should be referred emergently. Unstable SCFE have a much greater risk of avascular necrosis.

Treatment includes non-weight bearing, and surgery with epiphyseal fixation and possible osteotomy. Most patients do well after surgical fixation. Complications include avascular necrosis and chondrolysis. Patients require long term follow up as SCFE may develop within 12 to 18 months in the contralateral hip, if prophylactic pinning is not performed.[15]

Children who do not fit the typical profile for SCFE (under age 10 or over age 16, thin) should undergo evaluation for endocrinopathies (thyroid disease, growth hormone abnormalities) associated with SCFE.[16]

### Inflammatory and infectious disorders

#### The irritable hip – Transient synovitis and septic arthritis

A young child with an irritable hip poses a diagnostic challenge. Transient synovitis and septic arthritis have similar early symptoms with the spontaneous onset of progressive hip, groin, or thigh pain; limp or inability to bear weight; fever; and irritability. Transient synovitis typically has an acute onset, and spontaneous recovery with no radiological abnormality or systemic upset. It occurs between the ages of 2 and 10 years (peaking between 5 and 6 years) and is more common in boys, often preceded by viral infection. It is a self-limited condition with no recognized long-term sequelae and can be managed with oral analgesics and observation [17,18]. Transient synovitis recurs in up to 15% of children, and may affect the same or opposite hip.[19]

Children with septic arthritis appear ill and early management with surgical drainage and intravenous antibiotics is necessary to prevent bony destruction and preserve hip function. A septic hip is a surgical emergency. Hip aspiration is considered the gold standard for diagnosis of septic arthritis, with greater than 100,000 white blood cells per mm^3 ^with a predominance of polymorphonuclear cells or positive gram stain and culture confirming the diagnosis. Septic arthritis is rare if there are less than 25,000 white blood cells per mm^3 ^or less than 75% polymorphonuclear cells. In between these parameters is a grey zone and clinical prediction rules have been created to help aid diagnosis. CBC, ESR or CRP, blood culture and joint aspirate in BACTEC culture bottles should be sent immediately. Additional tests to consider depending on the history include Lyme serology, ANA, rheumatoid factor, HLA B27 and TB skin test.

A variety of clinical, laboratory, and radiographic criteria are used to help differentiate septic arthritis from transient synovitis, but because of the substantial overlap between the two groups there are no absolute criteria for definitive diagnosis of either condition. Kocher and colleagues described an Evidence-Based Clinical Prediction Algorithm to determine the probability of septic arthritis and subsequently validated the algorithm in a prospective study.[20] The four independent multivariate clinical predictors to differentiate between septic arthritis and transient synovitis are: history of fever, non-weight-bearing, erythrocyte sedimentation rate of at least 40 millimeters per hour, and serum white blood-cell count of more than 12,000 cells per mm^3 ^(12.0 × 10^9 ^cells per liter). The predicted probability of septic arthritis was determined for all sixteen combinations of these four predictors and is summarized (original cohort/prospective cohort) as less than 0.2 percent for zero predictors, 3.0/9.5 percent for one predictor, 40.0/35.0 percent for two predictors, 93.1/72.8 percent for three predictors, and 99.6/93.0 percent for four predictors.[20,21] In another preliminary retrospective study, Jung and colleagues found the probability of diagnosing acute septic arthritis was 98.8% according to multivariate regression analysis and the presence of all of the following five features: body temperature >37°C, ESR >20 mm/h, CRP >1 mg/dL, WBC>11,000/mL, and an increased medial hip joint space of >2 mm on A/P radiographs.[22]

Clinical prediction rules serves as guidelines but clinical judgment ultimately dictates patient management.

#### Osteomyelitis

Proximal femur or pelvic osteomyelitis presents similar to septic arthritis of the hip with fever and pain but children may have some passive range of motion if there is not extension of the infection into the joint. Osteomyelitis of the femur is common and usually occurs in the rapidly growing metaphyseal region.[23] Pelvic osteomyelitis is not common; it usually occurs in late childhood and affects the ilium (40%), ischium (28%) and the pubis (15%).[24] Pain may be referred to the hip, thigh, or abdomen, often leading to delays in diagnosis. Results of blood cultures are positive in less than 50% of patients. The most common pathogen in normal hosts is S. aureus. Technetium bone scan will localize the infection and CT or MRI imaging can better characterize the extent of involvement. Treatment includes initial intravenous antibiotics followed by oral antibiotics. If there is an abscess surgical drainage is required.

#### Pyomyositis

Pyomyositis is predominantly a disease of tropical countries and occurs more commonly in the warmer regions of a country and during the warmer months. Large muscle groups of the pelvic girdle and lower extremities are the most common sites of infection and deep muscle infections can mimic septic arthritis. Children present with fever; swelling of the affected muscle; minimal, if any, overlying skin changes; and guarded hip motion and pain with passive stretch of the involved muscle.[25] MRI imaging is the best imaging study for intramuscular abscesses. Treatment includes surgical drainage if there is an abscess and initial intravenous antibiotics followed by oral antibiotics.

#### Juvenile idiopathic arthritis (JIA)

Hip disease develops in 30–50% of children with JIA and is usually bilateral.[26] It is very uncommon for a child to present with hip monoarthritis as the initial manifestation of JIA. Exclusion of infection, including mycobacterium tuberculosis is very important. Children with hip arthritis usually present with groin pain but may have referred thigh or knee pain. The typical history of morning stiffness, gradual resolution of pain with activity and clinical exam findings of painful or decreased range of motion, especially in internal rotation usually allow the practitioner to make the correct diagnosis. A complete joint and systemic examination to exclude other joint involvement is important as is screening for asymptomatic uveitis associated with JIA. Initial radiographs may be normal or show regional soft tissue swelling and osteopenia. Ultrasound can assess effusions and pannus while MRI best assesses early disease findings of synovial proliferation and cartilage erosions.[27] Treatment includes physiotherapy for improving range of motion and strength, NSAID therapy, potential local intra-articular corticosteroid injection and disease modifying anti-rheumatic therapy. All children and adolescents suspected of having JIA or other chronic inflammatory arthritis should be referred to a pediatric rheumatologist.

#### Reactive arthritis

Reactive arthritis secondary to gastrointestinal and genitourinary pathogens is not common in children. Children who carry the HLA-B27 antigen are more likely to have a chronic course and involvement of the hips and sacroiliac joints in a spondyloarthropathy pattern. Treatment includes NSAID therapy, physiotherapy and disease modifying anti-rheumatic therapy in persistent cases.

#### Chondrolysis

Idiopathic chondrolysis of the hip is characterized by pain and limp in adolescence, with progressive loss of articular cartilage space by an undefined but presumably inflammatory process. Girls are affected more than boys and symptoms of pain and stiffness are commonly unilateral. There are no systemic features and investigations (hematological, microbiological, immunological and acute phase reactants) are normal. Early radiographs may be normal and late radiographs commonly show regional osteoporosis, premature closure of the femoral capital physis, narrowing of the joint space, and lateral overgrowth of the femoral head on the neck. MRI shows loss of articular cartilage with minimal synovial enhancement.[28] Some patients recover but many go on to develop painful and disabling osteoarthritis of the hip. Management includes protective weight bearing, NSAID therapy, physiotherapy and orthopedic intervention as necessary.[29]

### Overuse injuries and pain

#### Muscle Strains and Tendinopathies

Hamstring and quadriceps injuries are common in active young people, especially during the adolescent growth spurt when skeletal growth is greater than muscle-tendon-unit growth. Avulsion injuries must always be considered in skeletally immature adolescents. Muscle injuries include contusions, strains, tendinopathy and complete rupture. Adolescents usually present with localized pain which is aggravated by activity. On examination, there may be tenderness at the muscle attachment, musculotendinous junction or in the muscle belly; swelling or ecchymoses; pain with passive stretch and with resisted strength testing. Management includes rest, ice, compression, activity modification, and physiotherapy.

#### Apophysitis and apophyseal avulsion injuries

Apophyseal avulsion fractures are common in young athletes; affect boys more often than girls, and usually occur secondary to forceful or repetitive traction of the attached muscle. The apophyses of the pelvis appear and fuse later than the growth plates in long bones, placing adolescents at increased risk of avulsion injury, especially during a 'growth spurt'. Common sites of avulsions are at the iliac crest (abdominal muscles), anterior superior iliac spine (sartorius), anterior inferior iliac spine (rectus femoris), ischial tuberosity (hamstrings), and lesser trochanter (iliopsoas). (Figure 1) Adolescents usually present with localized pain, swelling and limited range of motion. Radiographs demonstrate displacement of the apophyseal center from its normal position, callus, and bony reaction. MRI is useful in suspected avulsion injuries with normal radiographs.[30] Management is usually non-operative and includes rest, ice, modified activity and physiotherapy. Most adolescents are able to return to their athletic activities within 4 to 8 weeks.

#### Stress fractures

Stress fractures of the femur are uncommon in children, but they can occur in athletes engaged in repetitive loading of the lower extremities such as endurance running.[31] Children present with activity related hip, groin or anterior thigh pain that can typically be reproduced by asking the patient to hop on the affected leg. Radiographs will detect chronic stress reaction. Early diagnosis or fractures not seen on x-ray can be diagnosed by technetium bone scan or MRI. Femoral neck stress fractures require special attention. Fractures through the inferior part of the femoral neck (compression side) can be treated with limitation of activity and weight-bearing. Fractures through the superior aspect (tension side) require surgical stabilization.

#### Snapping hip

Snapping hip syndrome refers to patient report of sounds or sensations of snapping or clicking with hip flexion and extension. A minority of children and adolescents will report pain, which is usually from the associated bursa. Children may have tenderness to palpation over the greater trochanter and posterior to it. An internal snapping hip relates to the iliopsoas bursa and an external snapping hip relates to the trochanteric bursa. An internal snapping hip is usually due to movement of the iliopsoas tendon over the iliopectineal eminence and less commonly over the lesser trochanter or ASIS. An external snapping hip is due to movement of the iliotibial band over the greater trochanter.[32] Several alignment and biomechanical factors are associated with a snapping hip including tight iliotibial band, narrow bi-iliac width, strength and flexibility imbalance and poor training techniques. Treatment involves identification of contributing factors, rest and avoidance of aggravating activities and targeted physiotherapy and rehabilitation. Children with refractory pain and trochanteric bursitis may benefit from a corticosteroid injection. Rarely, bursal excision and Z-plasty to lengthen the IT band may be required for recalcitrant cases of external snapping.[33]

### Tumors

Benign and malignant tumors can cause hip and groin pain. The proximal femur is a common site for benign osteoid osteomas. The classic symptom complex is bone pain at night and relief with NSAID's. Lesions may be visible on radiographs as lucencies with surrounding cortical thickening and sclerosis. Malignant tumors include local Ewing's sarcoma, soft tissue sarcomas, leukemia or metastases from neuroblastoma.

### Non musculoskeletal causes of pain

It is important to recognize referred pain from low back, intra-abdominal or pelvic pathology. In adolescent athletes groin pain can be difficult to diagnose and manage. "Athletic pubalgia" includes a spectrum of related pathologic conditions resulting from musculotendinous injuries and subsequent instability of the pubic symphysis.[34] Osteitis pubis (inflammation of the pubic symphysis), 'sportsman's hernia' (posterior inguinal wall weakness) and nerve entrapments (obturator, ilioinguinal, lateral femoral cutaneous nerve) can be difficult to evaluate. Adolescents typically present with poorly localized pain, aggravated by activity. Dysesthesia may be present in cases of nerve entrapment. An inguinal hernia is not found on physical examination. MRI of the pelvis and high-resolution MR imaging of the pubic symphysis may help to localize and define the extent of injury.[35] Referral to a sports medicine physician may help expedite appropriate diagnosis and management.

## Conclusion

Hip pain is a common complaint in children and adolescents. The clinical history and examination, age, gender and body habitus helps focus the differential diagnosis. It is important to recognize serious causes of hip pain and in particular to differentiate infection from transient synovitis. Radiographs are useful in the evaluation of a child with hip pain and can help identify the need for further imaging and referral.

## Competing interests

The author declares that they have no competing interests.

## Authors' informations

KH is certified in both pediatric rheumatology and sports medicine and has a Masters of Science in Human Kinetics.
